# *PLoS Biology* at 5: The Future Is Open Access

**DOI:** 10.1371/journal.pbio.0060267

**Published:** 2008-10-28

**Authors:** Theodora Bloom, Christine Ferguson, Liza Gross, Catriona J MacCallum, Jami Milton, Robert Shields, Stephanie Wai, Janelle Weaver, Liz Williams

## Abstract

Five years after*PLoS Biology* launched, the publishing landscape has changed radically. How much have PLoS and*PLoS Biology* contributed to this change, and what does the future hold for open access?

On the 13th of October in 2003, with the first issue of *PLoS Biology*, the Public Library of Science realized its transformation from a grassroots organization of scientists to a publisher. Our fledgling website received over a million hits within its first hour, and major international newspapers and news outlets ran stories about the journal, about science communication in general, and about our founders—working scientists who had the temerity to take on the traditional publishing world and who pledged to lead a revolution in scholarly communication (see, for example, [1,2]). It was not only scientists and publishers who wanted to see what this upstart start-up was doing; we had somehow captured the imagination of all sections of society. Not all of the reactions were positive, of course, especially from those in the scientific publishing sector with a vested interest in maintaining the subscription-based system of journal publishing. But thanks in no small part to the efforts of the founders—Pat Brown, Mike Eisen, and Harold Varmus—and an editorial team that included a former editor of *Cell* and several from *Nature*, our call for scientists to join the open-access revolution [3,4] did not go unheeded. Five years on, the publishing landscape has changed radically. How much have *PLoS Biology* and PLoS contributed to that change and what might the future hold for us and for publishing?

*PLoS Biology* is the flagship journal that gave PLoS its initial credibility as a publisher, paving the way for the equally successful launch of the flagship medical journal *PLoS Medicine*, four leading subject-specific journals (*PLoS Computational Biology*, *PLoS Genetics*, *PLoS Pathogens* and *PLoS Neglected Tropical Disease*), and its most radical, interdisciplinary peer-reviewed upstart, *PLoS ONE* [5].

By any traditional measure—authority of the editorial board, impact factor, professional staff editors, rejection rate, downloads, media attention, and so on—*PLoS Biology* is successful and has achieved that success rapidly. The proximate reason for our success lies in our establishing a high-quality journal that covers all aspects of biology, from molecules to ecosystems. This glib statement belies the fact that it has taken the commitment and dedication of our editorial board, our newly appointed Academic Editor-in-Chief Jonathan Eisen, and the courage of our pioneering authors to contribute to a new journal with an unproven publishing model because they believed in making the scientific literature a freely available public resource. And behind these visible individuals are the thousands of others who have reviewed and submitted articles, and who continue to do so. Without this remarkable community support, we would not be here. This groundswell of support is also the reason behind the most significant achievement of PLoS: not the journals themselves, but rather their larger impact on publishing, on funding agencies, and on scientific communication more generally.

The past five years have seen fundamental changes in the publishing infrastructure. Major funding bodies, including the National Institute of Health (NIH), the Wellcome Trust, the European and UK Research Councils, and many others, have mandated that the research they fund be made freely available (for a complete list, see [6]). Monthly submissions of NIH-funded articles to PubMed Central reached 4,000 in July this year (see [7] for the latest statistics). Institutions as well as funding agencies are mandating public access to the intellectual output of their researchers, and are developing alternative mechanisms to support payment of publication fees in open-access journals, public licensing of their articles, and archiving (including The University of California Berkeley [8], Harvard [9], and those listed in the Registry of Open Access Repositories (see Table 1 for a list of Websites associated with organizations or initiatives listed in this article). There are also now more that 3,600 journals listed on the Directory of Open Access Journals (DOAJ), and the list is growing at a rate of more than two per day [10]. And open access is driving a change in scholarly communication not just in the physical and life sciences but also in the humanities (e.g. The Open Humanities Press). Perhaps most telling of all is that many commercial and nonprofit publishers are experimenting with open access by providing open-access journals or an open-access option for their authors (although you should always read the small print carefully [11]).

**Table 1:**
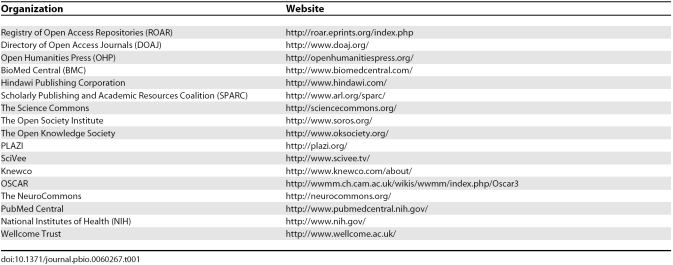
Websites of Organizations Listed in the Text

It is not possible to measure *PLoS Biology*'s or even PLoS's contribution to all this change. We are now a small part of a much larger movement. Open-access publishers, such as BioMed Central and Hindawi, were operating before PLoS and remain very influential in making open-access publishing a commercial and public success. There are numerous institutions, libraries, and advocacy organizations—such as the Scholarly Publishing and Academic Resources Coalition (SPARC), the Science Commons, the Open Society Institute, and the Open Knowledge Society —as well as individual advocates (most notably Peter Suber [12]) or specific science-led projects (e.g., “PLAZI” for taxonomists) operating worldwide to promote open access and public archiving of the literature. And it is the vision of funding agencies—in particular the Wellcome Trust and NIH—that is now forging the change toward open access most effectively. Perhaps the key contribution that *PLoS Biology* made in 2003, and, along with the other PLoS journals, has continued to make ever since, is to provide open-access forums where researchers can confidently publish world-class science.

The next challenge—for *PLoS Biology*, for PLoS and for all open-access publishers—is to demonstrate the utility of open access in advancing science beyond what can be gained from just making the information publicly available to read. The biggest misconception about open access is that it's only about putting online what was in print and removing any toll for access. It's not: it's about having the freedom to reuse that material without restriction [11]. Open-access publishing is therefore a crucial catalyst for a genuine shift in the way we use and mine the literature and integrate it with databases and other means of scientific communication. We are only just beginning to see the start of these: in video-based initiatives such as SciVee (Table 1); in knowledge discovery platforms such as Knewco, OSCAR, and the NeuroCommons (Table 1); with the increasing use of blogging in discourse about scientific research (see, for example, http://researchblogging.org/); and in the emergence of wiki projects in community-based knowledge curation [13,14].

As for the journal itself, *PLoS Biology*'s key goal remains essentially the same as it was for our first issue; to attract and publish outstanding papers in the broad field of biology. Our founders laid it out in their 2003 editorial [4]. “With all that is at stake in the choice of a journal in which to publish—career advancement, grant support, attracting good students and fellows—scientists who believe in the principle of open access and wish to support it are confronted with a difficult dilemma.” This challenge remains the case today because most open-access journals—even *PLoS Biology*—are still new and lack the prestige of established toll-access journals [15]. And, as Peter Suber notes, “it will take time for OA journals to earn prestige in proportion to their quality” [15].

Those of us who have taken part in the open-access scientific revolution can feel proud: open access has come far. But we must not be complacent. Most scientific publications still remain behind a subscription or other access barrier. For those who have not yet taken part, there is still time to help change the system. Commit to making your research-related publications open access by publishing in open-access journals and archiving your existing papers in publicly available digital repositories. It is not just the future—but your future—that is open access.
